# Dramatic increase in water use efficiency with cumulative forest disturbance at the large forested watershed scale

**DOI:** 10.1186/s13021-021-00169-4

**Published:** 2021-03-01

**Authors:** Krysta Giles-Hansen, Xiaohua Wei, Yiping Hou

**Affiliations:** University of British Columbia, 1177 Research Road, Kelowna, BC V1V 1V7 Canada

**Keywords:** Forest carbon, Hydrology, Cumulative disturbance, Water use efficiency, Forest carbon and water coupling, Large watershed, Evapotranspiration, Machine learning

## Abstract

**Background:**

Forest disturbance induced changes in the coupling of forest carbon and water have important implications for ecosystem functioning and sustainable forest management. However, this is rarely investigated at the large watershed scale with cumulative forest disturbance. We used a combination of techniques including modeling, statistical analysis, and machine learning to investigate the effects of cumulative forest disturbance on water use efficiency (WUE, a proxy for carbon and water coupling) in the 19,200 km^2^ Chilcotin watershed situated in the central interior of British Columbia, Canada. Harvesting, wildfire, and a severe Mountain Pine Beetle (MPB) infestation have gradually cumulated over the 45-year study period, and the watershed reached a cumulative equivalent clear-cut area of 10% in 1999 and then 40% in 2016.

**Results:**

Surprisingly, with the dramatic forest disturbance increase from 2000 to 2016 which was mainly due to MPB, watershed-level carbon stocks and sequestration showed an insignificant reduction. This resilience was mainly due to landscape-level carbon dynamics that saw a balance between a variety of disturbance rates and types, an accumulation of older stand types, and fast growing young regenerated forests. Watershed-level carbon sequestration capacity was sustained, measured by Net Primary Production (NPP). A concurrent significant decrease in annual evapotranspiration (ET), led to a 19% increase in WUE (defined as the ratio of NPP to ET), which is contrary to common findings after disturbance at the forest stand-level. During this period of high disturbance, ET was the dominant driver of the WUE increase.

**Conclusions:**

We conclude that disturbance-driven forest dynamics and the appropriate scale must be considered when investigating carbon and water relationship. In contrast to the stand-level trade-off relationship between carbon and water, forested watersheds may be managed to maintain timber, carbon and water resources across large landscapes.

## Background

People throughout the world rely on water supplied from forested watersheds [1], as well as other ecosystem services and values such as timber production or carbon sequestration [2, 3]. Forest carbon and water are coupled through the photosynthetic process and at the ecosystem level through carbon production and associated water consumption. This relationship is frequently altered by both natural and anthropogenic forest disturbance, which has a range of important implications for ecosystem structure, functions and services [4–9].

Water use efficiency (WUE), the ratio of carbon uptake to water use, has been commonly used as a proxy to study the relationship between forest carbon and water in forest ecosystems. WUE is used to evaluate the potential impacts of climate change on food production [10, 11], water supply [7], and forest or land use management [12–14]. WUE has been studied from the leaf to global scale, so a variety of definitions are found in the literature. Studies looking at leaf-level WUE concentrate on measuring the ratio of net CO_2_ assimilation to stomatal conductance at sub-daily time scales [15]. Tree-level WUE can be measured through isotope analysis, sap-flow measurements [16], or controlled environment chamber experiments [17] at time scales from daily to seasonal or annual [18]. Stand-level WUE includes the additional effects of other vegetation and evaporation, and water and carbon exchange is often measured using eddy-covariance systems [19, 20]. Watershed or landscape-level studies have involved the use of modeling or analysis based on the water budget [21, 22]. Regional to global studies of WUE often rely on modeling or remote sensing [13, 14, 23], comparing trends across global gradients or investigating the impact of climate change. Among the existing studies on WUE at various spatial scales, large watershed- or landscape-level studies are rare, likely due to the difficulty of conducting field measurements [24].

Disturbance can change stand-level WUE [25]. However, studies are relatively limited with the body of research focusing on how drought or climate change affects WUE [12, 26, 27]. The majority of research into WUE after forest disturbance is at the stand-level using eddy covariance data. These studies found that severe forest disturbance often results in a decrease in WUE, most likely because of greater relative surface evaporation [28], while lower intensity disturbance such as insect attack or prescribed burning can also produce a reduction or no change in WUE [25, 29, 30]. There is an emerging view that the effect of forest disturbance on WUE is variable, depending on tree mortality, site conditions, remaining vegetation recovery, and time since disturbance. In addition to the site-level nature of most WUE measurements, another limitation is that study periods usually cover just the few years following disturbance. In large watersheds or landscapes, forest disturbances of different types cumulate over space and long periods of time, and consequently their effects on forest carbon, water and WUE may be different. To our knowledge there are no measurement-based studies and few modeling-based studies that have examined the effects of long term cumulative forest disturbance on WUE at the large watershed-level.

To advance this topic in the literature, we investigated the forest carbon–water relationship in the Chilcotin watershed situated in the interior of British Columbia, Canada, a large forested watershed that experienced high level of cumulative forest disturbance over the last 45 years. We hypothesise that cumulative forest disturbance is one of the key drivers of carbon, water and WUE. We expect that WUE decreased under significant cumulative forest disturbance in this large forested watershed, in a similar manner found at the forest stand or small watershed scale.

## Methods

### Study area

**Fig. 1:**
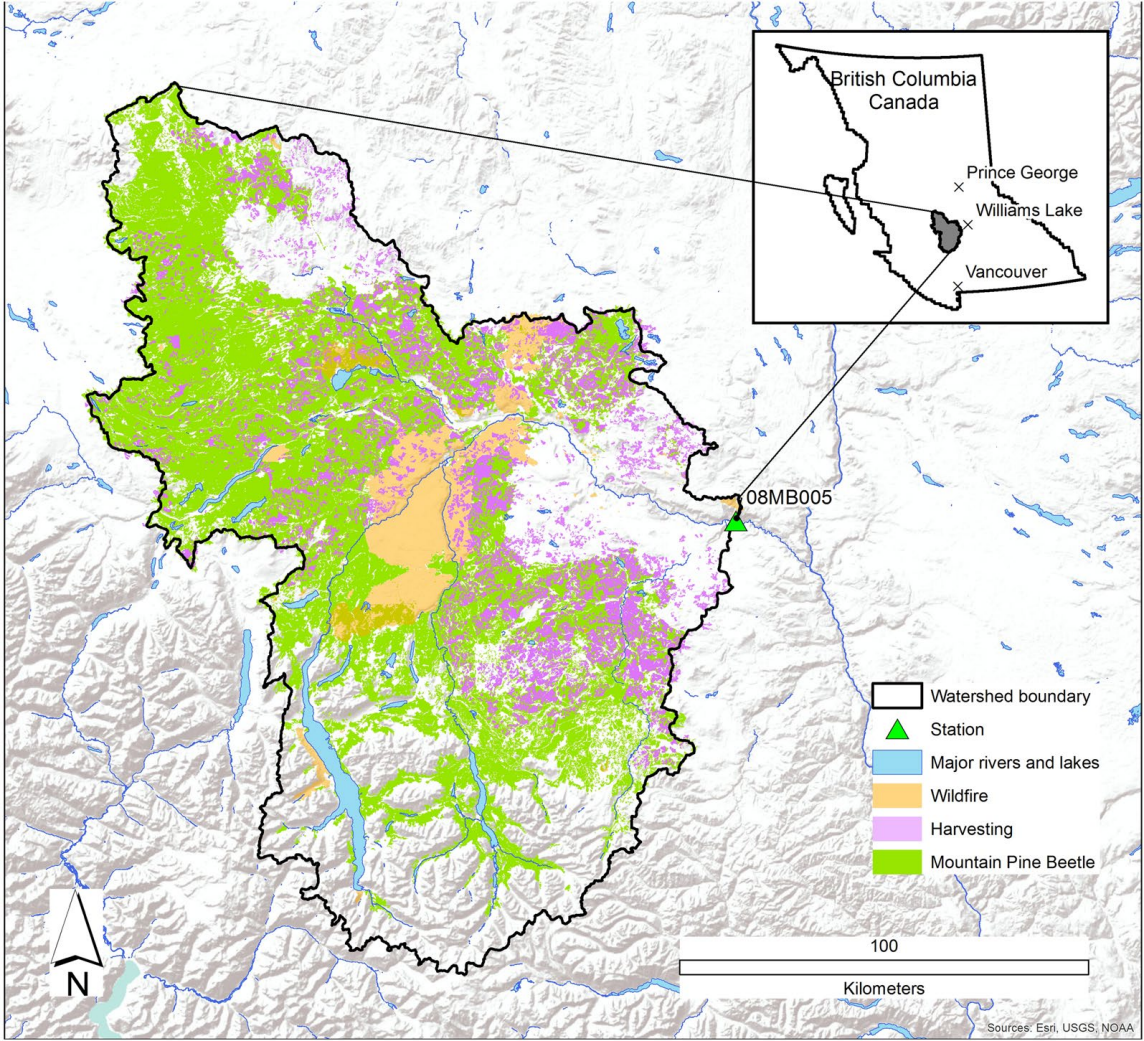
Location of the Chilcotin watershed in the central interior of British Columbia, Canada

The Chilcotin watershed has a drainage area of 19,200 km^2^ and is located 50 km west of Williams Lake on the Fraser plateau in British Columbia, Canada (Fig. 1). The topography is mountainous, ranging from 2,800 m above sea level (m) on the south western-end of the watershed in the coastal mountains down to 500 m in the lower reaches in the east. The large elevation gradient means ecosystems range from non-treed dry Bunchgrass Very Dry Warm Alkali variant (BGxw2) biogeoclimatic (BEC) zone in the valley bottoms up to Coastal Mountain-heather Alpine (CMAunp) BEC zone characterised by alpine tundra [31]. The majority of the watershed is forested in the Interior Douglas-fir (IDF), Sub-boreal Pine–Spruce (SBPS), and Montane Spruce (MS) BEC zones. These are dominated by *Pinus contorta Douglas ex Loudon* (Lodgepole pine) (78%) with significant components of *Pseudotsuga menziesii (Mirb.)* Franco (Douglas fir) (9%) and *Picea engelmannii Parry ex Engelm* (White spruce) (8%) forests, and minor components of other species.

Wildfires occur regularly in the study area and ecosystems are adapted to frequent stand-initiating and stand-maintaining fires. The provincial land has supported sustained levels of clear-cut logging since the 1960s. The Mountain Pine Beetle (MPB) is endemic throughout the region and a widespread severe infestation caused the mortality of a large proportion of mature Lodgepole pine trees in British Columbia in the early 2000s.

The climate is continental with an average winter temperature of − 8 °C and 11 °C in the summer. Annual precipitation (P) averages 635 mm which is distributed throughout the year, with a slightly lower proportion in the spring and summer months (19 and 21% respectively). Winter P falls as snow in all but the lowest valley bottoms, leading to a pronounced freshet with the spring melt in May or June.

### Data

#### Climate and streamflow data

**Fig. 2:**
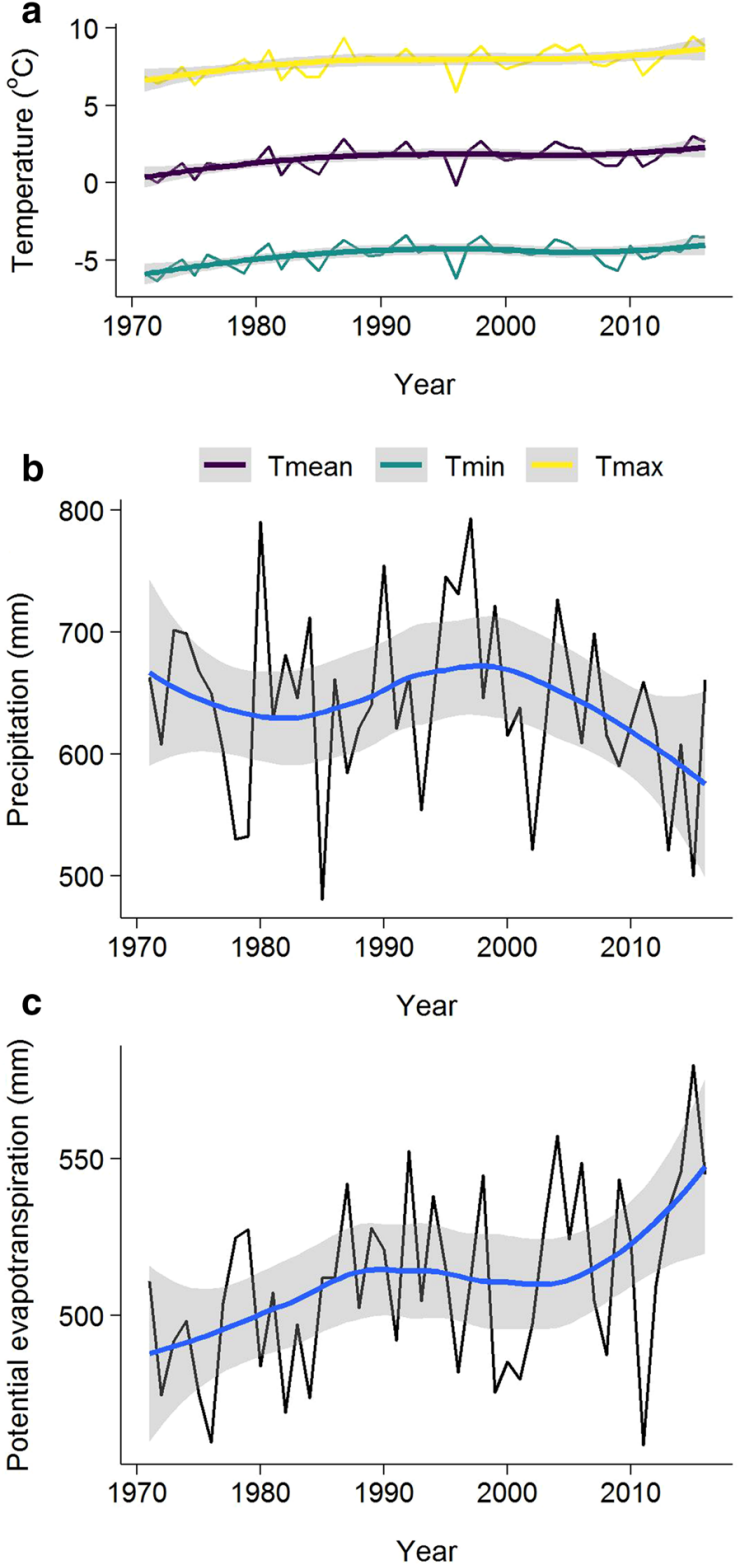
Annual climate variables in the Chilcotin watershed from 1971–2016. Where, **a** minimum (Tmin), mean (Tmean), and maximum (Tmax) daily annual temperature (**c**), **b** annual precipitation in millimeters (mm), and **c** potential evapotranspiration calculated using the Hargreaves equation

Annual timeseries of water, climate, carbon, and forest disturbance data were constructed based on streamflow data availability from 1971 to 2016. The annual climate variables, precipitation (in mm), annual mean, minimum and maximum temperature (Tmean, Tmin, and Tmax) were calculated using the ClimateBC dataset [32] based on a 25 m resolution digital elevation model [32, 33] and were averaged to obtain one estimate for the watershed. Potential evapotranspiration (PET), based on the ClimateBC temperature data was calculated using the Hargreaves method [34] (Fig. 2). Daily discharge was acquired from Water Survey of Canada (station ID: 08MB005) [35], as well as the corresponding drainage area [36]. The average of all daily flows in m^3^ sec^−1^ was used to calculate mean annual streamflow (Q), standardized to millimeters per year (mm year^−1^) based on watershed area. Runoff ratio was calculated as annual Q/P.

#### Forest disturbance

Forest disturbance information was sourced from two spatial layers maintained by the provincial government. The Vegetation Resources Inventory (VRI) (version 2019) contains the type, year and severity of the most recent logging and non-logging disturbance of each polygon [37]. Additionally, the provincial Consolidated Cutblocks (version 2019) layer was used to capture any recent logging that may have not been updated in the 2019 VRI [38].

**Fig. 3:**
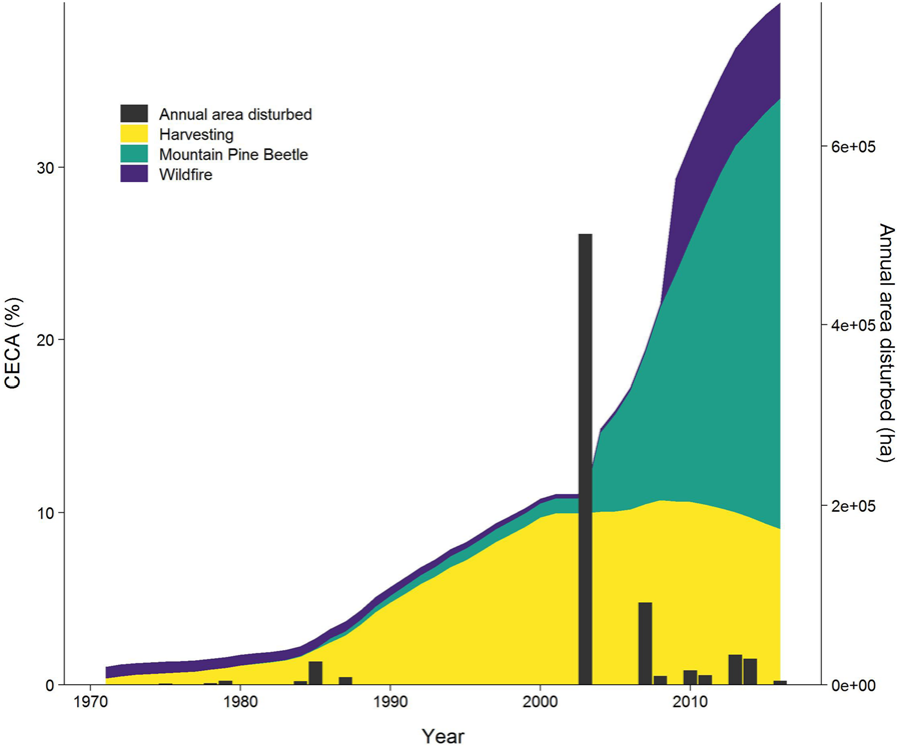
Chilcotin watershed Cumulative Equivalent Clear-Cut Area (CECA) total annual area disturbed from 1971–2016. Where, CECA is colored by disturbance type (left axis), and grey bars represent total annual area disturbed in hectares (ha) from all types of disturbances (right axis)

Cumulative equivalent clear-cut area (CECA) was chosen as the watershed-level indicator of forest disturbance [39, 40]. CECA represents the cumulative effect of multiple distinct types of disturbance and recovery over time as vegetation re-grows. CECA has been widely used in British Columbia where winter snowpack and the effects of vegetation alteration on peak flows are a major concern [41–44]. Stand-level annual equivalent clear-cut area (ECA) is calculated as disturbed area multiplied by a coefficient, which ranges from 0 to 100%. The ECA coefficient is set at 100% after harvesting and wildfire, to reflect changes in hydrological processes such as infiltration and evapotranspiration (ET) and is reduced as the forest recovers [45]. After MPB mortality, an ECA coefficient is applied gradually as tree death and needle drop occur progressively over many years [46, 47]. In this study, hydrological recovery starts around 30 years after MPB mortality (see Additional file 1: Figure S1). In the years after disturbance, each polygon’s ECA score within the watershed was calculated based on the type of disturbance and years since disturbance. All ECA values in the watershed were summed and divided by the gross watershed area to calculate the total percent CECA for the watershed (Fig. 3). The Chilcotin watershed was essentially undisturbed at the start of the study period in 1971 with a CECA of 1%. Low levels of harvesting (an average of ~ 4,500 ha or 0.2% per year) dominated the disturbance profile until 2000 when the CECA reached 10%. From 2000 to 2016, forest mortality caused the CECA to increase to 39.6%, primarily from the MPB epidemic which affected just over 700,000 ha or 36% of the watershed to varying degrees (Fig. 3). Based on this disturbance timeline, we divided the study into two periods—from 1971 to 1999 which was characterised by a low rate of harvesting and a CECA <  = 10%, and from 2000 to 2016 where MPB, wildfire, and harvesting occurred at a higher rate (CECA from > 10–40%).

### Methods

#### Estimation of evapotranspiration

We used the Budyko–based Zhang’s equation (Eq. 1) to calculate annual watershed-level ET [48–50]. Zhang’s equation contains the *w* parameter that reflects the forest’s ability to transpire water at the watershed scale. We varied *w* to represent the change in ET with forest disturbance. To obtain independent estimates of *w*, we developed a simple linear regression equation between *w* and CECA from watersheds with similar topography and climate in the interior of British Columbia. Groups of five to 10 years with low, moderate, and high CECA values were chosen in the Chilcotin, Chilko, Baker, and Moffat watersheds. Annual P, PET, and Q were based on data ClimateBC [32] and Water Survey of Canada [35], and were calculated consistent with the description above. ET was calculated using the water balance equation (Eq. 2), where the change in storage over these five to 10 year grouped periods was assumed to be zero, thereby minimising the error associated with annual variations in storage. We then used the ‘uniroot’ function from the ‘rootSolve’ package [51] in R [52] to solve for *w*. Finally, using data from all four watersheds, we developed a linear equation to calculate *w* based on CECA (Additional file 1: Figure S3). This equation was used to calculate annual *w* for the Chilcotin watershed, which was then used along with annual P and PET in Eq. 1 to calculate annual ET in the Chilcotin watershed. We used this methodology to conceptually represent the change in ET from forest disturbance, however it still dependent on the accuracy of estimating precipitation over large areas. The water balance method showed similar trends, as did using three and five year averages, which gave us more confidence in the ET estimate calculated using Zhang’s equation (Additional file 1: Figure S4, Table S4 and Table S5). In this way, watershed level data has been used to scale point estimates of P partitioning to ET to the watershed [53].

1 $$\frac{ET}{P}=\frac{1+w\frac{PET}{P} }{1+w\frac{PET}{P}+{\left(\frac{PET}{P}\right)}^{-1}}$$

where, ET = evapotranspiration in mm, P = precipitation in mm, *w* = plant-available water coefficient (unit-less), and PET = potential evapotranspiration in mm, at the annual timescale.

2 $$P=Q+ET+\Delta S$$

where, P = annual precipitation, Q = mean annual streamflow, ET = annual evapotranspiration, and ∆S = annual change in storage, which was assumed to be zero.

#### Carbon modeling and validation

The Carbon Budget Model of the Canadian Forest Service version 3 (CBM-CFS3) [54, 55] was used to simulate annual historical carbon dynamics in the Chilcotin watershed. CBM-CFS3 is a yield curve based model widely used in Canada to simulate terrestrial carbon pools and their fluxes with the atmosphere [56, 57]. Forest information including age, disturbance history, species composition, and the productivity of the forest from the VRI [37], form the main inputs into the carbon modeling.

**Fig. 4:**
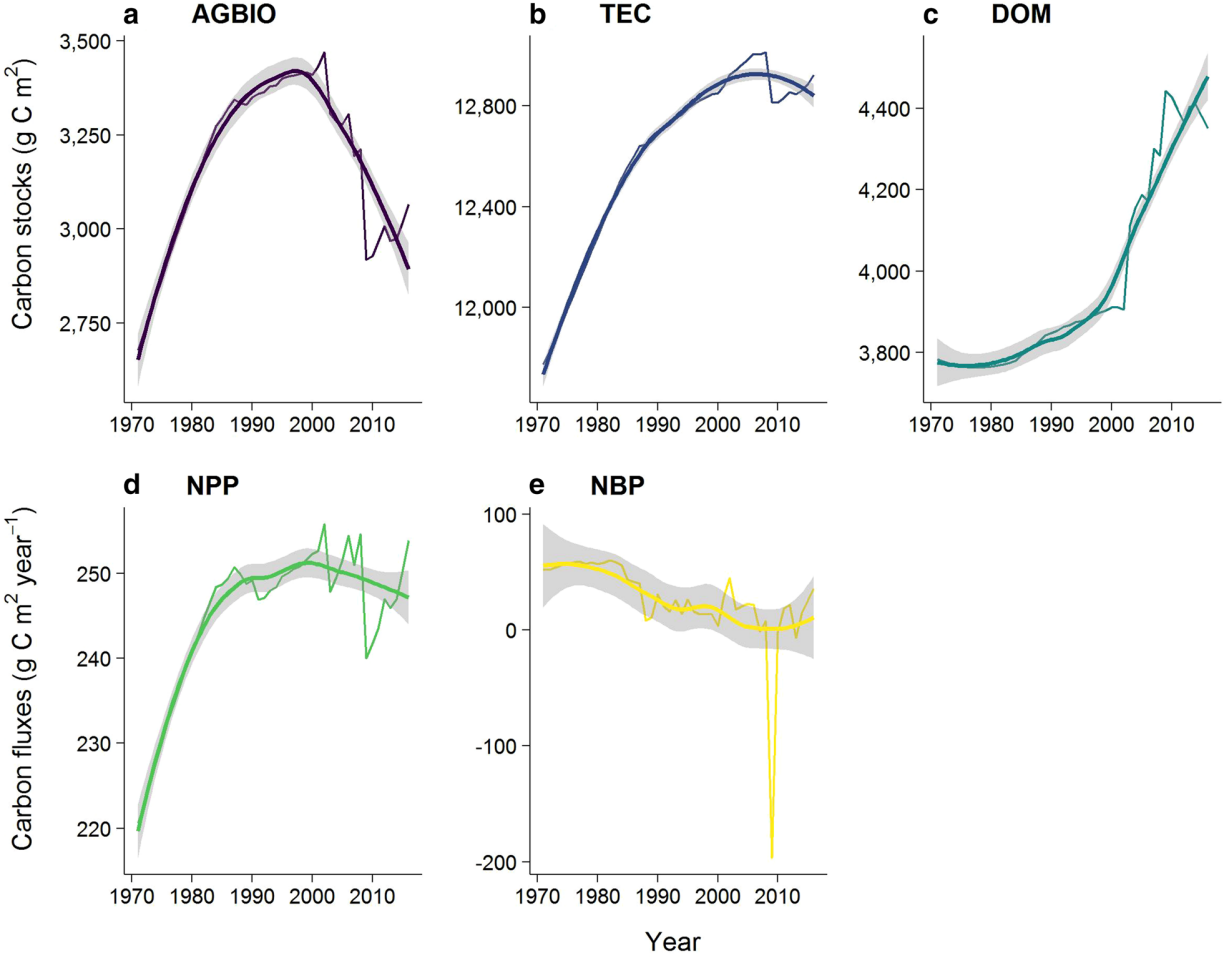
Watershed-level carbon variables in the Chilcotin watershed from 1971–2016. Where carbon stock pools are expressed in grams of carbon per square meter (g C m^−2^) and **a** is above ground biomass (AGBIO), **b** total ecosystem carbon (TEC), **c** dead organic matter (DOM), and carbon fluxes are in grams of carbon per square meter per year (g C m^−2^ year^−1^) **d** net primary production (NPP), **e** net biome production (NBP)

For stands with no disturbance or non-stand replacing MPB disturbance (mortality < 90%), the forest age in 1971 was calculated as the age in 2019 minus 49 years. For stands with disturbance that resets the stand age (wildfire, MPB with >  = 90% mortality, or harvesting), the VRI doesn’t record the previous stand type, so in order to fill this information gap, we populated these areas with the average of the non-disturbed stands by BEC. Yield curves were calculated using standard provincial growth and yield models. The Variable Density Yield Projection (VDYP) model uses VRI data to project yield curves for stands of natural origin. The Table Interpolation Program for Stand Yields (TIPSY) program was used to calculate yield curves for stands after harvesting. Disturbance types and other CBM-CFS3 parameters are detailed in Additional file 1: Table S2. Historical disturbances were explicitly scheduled in the CBM-CFS3 model [58], and the stand-level outputs were summed up to the watershed scale. We calculated the following watershed-level carbon variables: above ground biomass (AGBIO), dead organic matter (DOM), total ecosystem carbon (TEC), net primary production (NPP), and net biome production (NBP) [54, 55, 58–60] as shown in Fig. 4. CBM-CFS3 queries and definitions are shown in Additional file 1: Table S3.

CBM-CFS3 is a data driven model, with a key input being gross merchantable yield curves. CBM then applies allometric equations to estimate biomass and turnover parameters for dead organic matter and soil carbon [55]. We used 167 measurements of net merchantable volume from the provincial ground plot database [61] throughout the Chilcotin watershed, to validate our yield curves as a proxy for carbon stocks as in Smiley et al. [62]. We found good agreement between the modelled and measured values, where the modelled volumes average 95% of the plot data (R^2^ = 0.94, P < 0.001) (Additional file 1: Figure S7).

#### Calculation of WUE

**Fig. 5:**
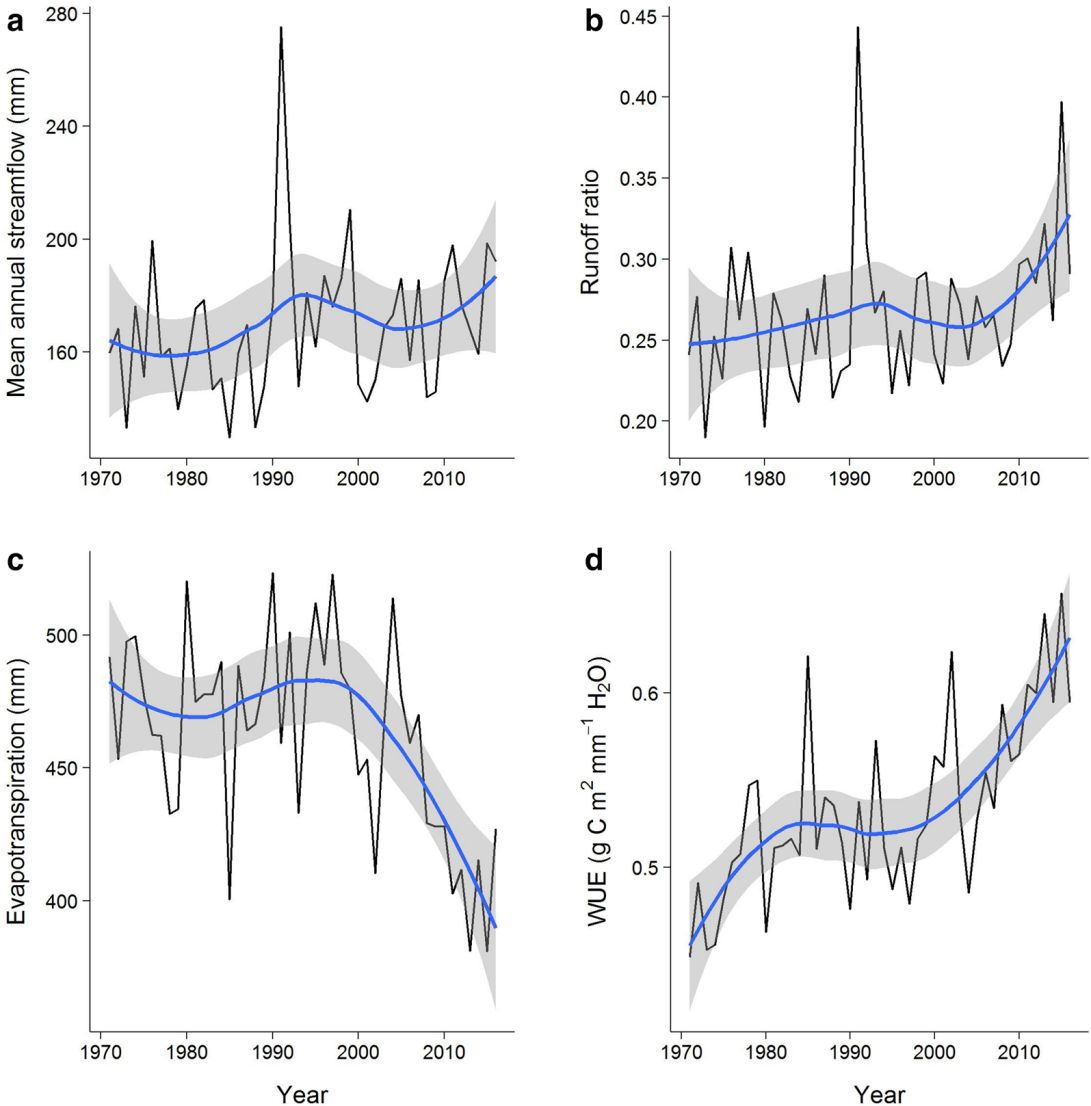
Annual water related variables in the Chilcotin watershed from 1971–2016. Where **a** mean annual streamflow in millimeters (mm), **b** runoff ratio, **c** evapotranspiration (ET) in mm, and **d** watershed scale annual water use efficiency (WUE) in grams of carbon per square meter per millimeter of water (g C m^−2^ mm^−1^ H_2_O), and the blue line is the loess regression smoothed line with 95% confidence limits shown as grey shading

The average annual watershed-level WUE was calculated as the ratio of NPP to ET in grams of carbon per millimeter of water per year (g C m^−2^ mm^−1^ H_2_O year^−1^) (Eq. 3, Fig. 5).

3 $$WUE= \frac{NPP}{ET}$$

#### Trend analysis

The non-parametric Mann–Kendall test [63] was implemented on pre-whitened data, following the process recommended in Yue et al. [64]. Although often used in hydrologic analysis [65–67], some recent studies have debated its applicability to non-stationary hydrologic data [68, 69]. However, we consider its use appropriate as it is one of multiple statistical tests used to help interpret the data. Trend analysis was applied separately to the periods of low and high cumulative disturbance to enable the detection of opposite monotonic trends in each period. All statistical tests are at a significance level of 5%.

#### Correlation analysis

Two rank-based non-parametric tests (Kendall correlation and Spearman correlation) [70] were used to test the direction and strength of the relationship between forest disturbance and carbon or hydrological variables. As in the trend analysis, they were applied separately to each period.

#### Gradient boosting machine

The tree-based machine learning Gradient Boosting Machine (GBM) method was used to investigate the relative importance of possible drivers in water and WUE variables. The ‘caret’ package in R [52, 71] was used to fit a GBM model for each variable. Data was pre-processed to scale predictor variables between 0 and 1. Highly correlated variables were removed based on the ‘cor’ function in the ‘caret’ package with a cut off of 90%, resulting in Tmean being dropped from the list of potential GBM model predictor variables. The optimal number of variables for each model was selected using the ‘rfeControl’ function in the ‘caret’ package that minimized the Root Mean Square Error (RMSE). 15 fold cross validation repeated three times was used to tune the GBM models, select the one with the lowest RMSE, and estimate model performance in terms of RMSE and R^2^. While not a strictly independent estimate of model accuracy, repeated cross validation is commonly used for machine learning models, particularly when an independent dataset is not available [72, 73]. Consistent with other studies, we reported on the relative importance of each predictor variable, the RMSE, and R^2^ of each final model. We ran four GBM models in total, one each for Q, ET, runoff ratio, and WUE with independent climate and forest disturbance variables (CECA, Tmax, Tmin, P, and PET) being the predictor variables in all cases.

#### Drivers of WUE variation

We calculated the magnitude of change in WUE and its components (NPP and ET) using Sen’s slope and Eq. 4 following El Masri [74]. The percentages of change in WUE, NPP, and ET were calculated for the low (1971–1999) and high (2000–2016) disturbance periods using the smoothed line from the loess regression (Figs. 4 and 5).

4 $$Change (\%)=100 \times \frac{y \times s}{m}$$

where, y = number of years in period, s = slope from the Sen’s slope test, m = mean value of NPP, ET, or WUE over the period.

## Results

### Trends in climate, carbon, and water

Forest disturbance, as measured by CECA significantly increased in the Chilcotin watershed during both the period of low disturbance and high disturbance, increasing from 1% in 1971 to 39.6% in 2016 (Fig. 3, Table 1). Contrary to our expectations based on stand-level dynamics, with increasing disturbance carbon stocks (AGBIO, TEC, and DOM) all significantly (P < 0.001) increased over the period from 1971 to 1999. Also over this first period, NPP increased significantly while NBP decreased or became more negative (P < 0.001). In the period of high disturbance from 2000–2016, AGBIO decreased significantly (P < 0.001) and DOM increased significantly (P < 0.001). Interestingly, TEC, NPP and NBP did not have any statistically significant trends in the high disturbance period.

**Table 1:** Time-trend analysis of annual variables in the Chilcotin watershed by period

Type of variable	Variable	1971—1999		2000—2016	
		Tau	P-value	Tau	P-value
Forest disturbance	CECA	1.000	< *0.001*	0.950	< *0.001*
Carbon	AGBIO	0.995	< *0.001*	− 0.750	< *0.001*
	TEC	1.000	< *0.001*	− 0.267	0.163
	DOM	0.995	< *0.001*	0.783	< *0.001*
	NPP	0.974	< *0.001*	− 0.217	0.260
	NBP	-0.799	< *0.001*	− 0.133	0.499
Climate	Tmean	0.280	*0.038*	0.200	0.300
	Tmax	0.280	*0.038*	0.217	0.260
	Tmin	0.302	*0.026*	0.067	0.753
	P	0.127	0.353	− 0.083	0.685
	PET	0.206	0.128	0.233	0.224
Water	ET	0.164	0.228	− 0.417	*0.027*
	Q	0.185	0.173	0.333	0.079
	Runoff ratio	0.037	0.797	0.150	0.444
Carbon and water relationship	WUE	0.032	0.828	0.383	*0.043*

Where, tau is the z-statistic from the Mann–Kendall test indicating the direction of change of the variable; p-value is the level of significance from the Mann–Kendall test; and bolded italics indicate significant trends at a significance level of 0.05), *CECA* cumulative clear-cut area, *AGBIO*  above ground biomass, *DOM*  dead organic matter, *TEC* total ecosystem carbon, *NPP*  net primary production, *NBP* net biome production, *Tmean* annual average daily temperature, *Tmin* annual minimum daily temperature, *Tmax*  annual maximum daily temperature, *P*  precipitation, *PET*  potential evapotranspiration, *ET* evapotranspiration, *Q* mean annual streamflow, *WUE* water use efficiency

Temperature increased significantly in the Chilcotin watershed from 1971 to 1999, but not in the period of high disturbance from 2000 to 2016. Tmean increased from an average of 0.86 °C to 1.82 °C in the first and last decade of the study period. P and PET did not have any statistically significant trend in either period. Of the hydrological variables, only ET had a significant negative trend (P = 0.027) from 2000 to 2016. WUE significantly increased from 2000 to 2016 (P = 0.043) (Table 1).

### Forest disturbance and carbon

Unexpectedly, the correlation tests for the period of high disturbance from 2000–2016 showed that total carbon stocks (TEC) and carbon sequestration (NPP) were not significantly related to CECA (Table 2). The lack of the significant correlations between CECA and carbon stock and sequestration variables, along with their insignificant trends clearly suggests that the severe cumulative disturbance from 2000 to 2016 did not cause significant reduction in carbon stock and sequestration. However, AGBIO was significantly negatively correlated with CECA (Table 2), while DOM had a significant and positive correlation with CECA from 2000–2016.

**Table 2:** Kendall and Spearman correlation results between forest disturbance and selected carbon and water variables

Type of variable	Variable	1971–1999				2000–2016			
		Kendall		Spearman		Kendall		Spearman	
		Cor	P	Cor	P	Cor	P	Cor	P
Carbon	AGBIO	0.99	*< 0.001*	1.00	*< 0.001*	− 0.54	*0.002*	− 0.78	*0.000*
	TEC	1.00	*< 0.001*	1.00	*< 0.001*	− 0.03	0.903	− 0.30	0.235
	DOM	0.77	*< 0.001*	0.88	*< 0.001*	0.62	*<0.001*	0.81	*<0.001*
	NPP	0.81	*< 0.001*	0.90	*< 0.001*	− 0.22	0.236	− 0.42	0.098
	NBP	− 0.52	*< 0.001*	− 0.75	*< 0.001*	− 0.07	0.715	− 0.10	0.708
Water	ET	0.15	0.271	0.22	0.244	− 0.46	*0.010*	− 0.62	*0.010*
	Q	0.27	*0.044*	0.39	*0.037*	0.37	*0.042*	0.53	*0.032*
	Runoff ratio	0.12	0.381	0.17	0.382	0.41	*0.022*	0.58	*0.016*
Carbon and water relationship	WUE	0.27	*0.044*	0.35	0.061	0.43	*0.017*	0.57	*0.018*

Where *Cor*  correlation coefficient, P is the level of significance, bolded italics indicate significant trends at a significance level of 0.05, *CECA*  cumulative clear-cut area, *AGBIO*  above ground biomass, *DOM*  dead organic matter, *TEC*  total ecosystem carbon, *NPP*  net primary production, *NBP*  net biome production, *Tmean*  annual average daily temperature, *Tmin*  annual minimum daily temperature, *Tmax*  annual maximum daily temperature, *P*  precipitation, *PET*  potential evapotranspiration, *ET*  evapotranspiration, *Q* mean annual streamflow, *WUE* water use efficiency

### Forest disturbance and water

Correlation analysis from the period of high disturbance found that all water variables had a significant correlations with forest disturbance (CECA). ET had a significant (P = 0.010) negative correlation with CECA from 2000–2016 with correlation coefficients of – 0.46 and – 0.62 for the Kendall and Spearman tests, respectively (Table 2). Both Q and runoff ratio showed significant (P < 0.05) positive correlations with CECA in the high disturbance period.

P was the most important variable in the runoff ratio and ET GBM models (R^2^ >  = 0.80), with relative importances of 41.6%, and 70.9% respectively (Table 3). The runoff ratio GBM model did not depend on CECA to explain variation. In contrast, P contributed 70.9% towards the prediction of ET in the GBM model, with CECA contributing the rest of the 29.1%. For the Q GBM model, the predictor variable with the highest importance was Tmin with 30.5%. P was a close second at 25.7%, while CECA accounted for 16.8%. In the Q GBM model, all climate variables contributed, reflecting the complex climatic and biological interactions and their influences on Q.

**Table 3:** Gradient Boosting Machine model results for selected variables

Variable	R^2^	RMSE	Relative importance of independent predictor variables: forest disturbance and climate				
			CECA	Tmax	Tmin	P	PET
Q	0.80	8	16.8	11.6	30.5	25.7	15.5
Runoff ratio	0.86	0.03	0.0	11.3	30.3	41.6	16.8
ET	0.90	29	29.1	0.0	0.0	70.9	0.0
WUE	0.93	0.04	44.4	0.0	0.0	55.6	0.0

Where each predictor variable’s relative importance ranges from 0 to 100, *CECA* cumulative clear-cut area, *Tmean*  annual average daily temperature, *Tmin  *annual minimum daily temperature, *Tmax *annual maximum daily temperature, *P*  precipitation, *PET* potential evapotranspiration, *ET*  evapotranspiration, *Q* mean annual streamflow, *WUE* water use efficiency, and *RMSE*  root mean square error

### Forest disturbance and water use efficiency

WUE averaged 0.53 g C m^−2^ mm^−1^ H_2_O over the study period and was significantly (P = 0.017, 0.018) positively correlated to CECA from 2000—2016, with correlation coefficients of 0.43 and 0.57 for the Kendall and Spearman tests, respectively. The WUE GBM model (R^2^ = 0.93, RMSE = 0.04, n = 45) placed importance on P (55.6%) and CECA (44.4%), reflecting the predictor variables that were relatively important to NPP and ET. In the period of low disturbance (1971—1999), WUE increased by 7% (Sen's slope P = 0.0001). This was largely attributable to increases in NPP which increased by 11%, compared to a 2% increase in ET. In the period of high disturbance (2000–2016) WUE increased by 19% (Sen's slope P < 0.001) driven primarily by a sharp reduction in ET. ET was reduced at a higher rate (− 19%) than NPP (− 2%) causing WUE to increase during this period.

## Discussion

### The effects of cumulative forest disturbance on carbon stocks and sequestration

Our study showed that the severe cumulative disturbance from 2000 to 2016 did not cause significant reduction in TEC stocks, but it significantly changed the AGBIO and DOM carbon pools. The significant reduction in AGBIO in the high disturbance period is consistent with both stand-level and landscape-level studies of carbon stock after disturbance [75–77]. Watershed-level DOM increased significantly after the high levels of MPB mortality in the early 2000s, as MPB killed living biomass in trees, converting it to DOM [55]. This suggests that there is a relationship between forest disturbance and some carbon sub-pools, and the conversion from AGBIO to DOM caused by MPB infestation was the key reason for insignificant reduction in the total amount of carbon stored in the watershed (TEC) despite severe cumulative disturbance. The CBM-CFS3 model does not explicitly account for the transfer of dissolved organic matter from the soil into rivers, which may be an important physical process to investigate further in relation to increasing disturbance [78, 79].

Studies have shown that forest disturbance can affect and drive changes in ecosystem services such as carbon sequestration and water provisioning [5]. Timber production and forest carbon are often presented as a trade-off relationship, with harvest driving lower carbon stocks or vice versa [2, 77]. At the stand-level, this is most certainly the case. However, this study showed that this relationship may not hold at the large watershed-level. Both the initial state of the watershed’s forest, and the slow rate of accumulation of forest disturbance until the early 2000s, allowed substantial TEC carbon stocks to persist despite disturbance. In areas with stand replacing natural disturbance regimes, harvesting may produce desired outcomes for carbon storage [80], especially if the carbon stored in harvested wood products are considered [81].

Interestingly, our analysis also showed that the cumulative disturbance from 2000 to 2016 did not cause significant reduction in carbon sequestration capacity of the watershed. This result is contradictory to the simple expectation that more disturbance equals lower NPP, a rule that generally holds at the site level, but may not be linearly extrapolated to large areas with significant variation or for all types of disturbance. Eddy covariance studies after MPB mortality have shown a resilience in NPP attributable to residual treed and non-treed vegetation [82, 83]. Kurz et al. [75] predicted an 11% drop in NPP and that the MPB epidemic would cause British Columbia’s central interior forests to be a carbon source at least until the year 2020 (NBP < 0). In the Chilcotin watershed however, the impact might not be severe enough to cause the watershed to be a substantial carbon source (average NBP from 2000–2016 was 4.43 g C m^**−**2^ year^−1^), except for 2009 when wildfires in the central part of the watershed emitted carbon (Fig. 4). Nevertheless, the average NPP in the disturbance period of 247.5 g C m^**−**2^ year^−1^ is within the range of what other modeling studies in the interior of British Columbia have found (NPP ranging from 118 to 660 g C m^**−**2^ year^−1^) [75, 84, 85].

There are two main reasons for non-reduction of carbon sequestration in the watershed as described above. Firstly, based on our knowledge of stand-level carbon dynamics after disturbance, we would expect NPP to decrease with increasing levels of disturbance [76, 77, 83, 86–90]. However, this stand-level relationship cannot simply be extrapolated to the watershed-scale over long periods of time because of forest recovery from regenerated forests or understory vegetation of the MPB stands. The type of disturbance and stand productivity determine the rate of recovery, with NPP recovering to pre-disturbance levels by an average of 20 years after harvesting (Additional file 1: Figure S2). Secondly, the low and consistent levels of harvesting that occurred in the watershed converted a portion of the watershed from older forest to young faster growing stands. In British Columbia, all harvested stands must be replanted after harvesting, often with control of competing vegetation, seedling spacing, and seed selection to enhance growth rates [91]. The cohort of young, fast growing forest has the effect of increasing the overall growth rate (or NPP) of the watershed. This finding is similar to other retrospective studies in British Columbia that found carbon uptake in recovering young forests was able to partially offset disturbance emissions [57] and that younger forests had higher CO_2_ uptake [80].

### Cumulative forest disturbance and water processes

Our results showed that the partitioning of P into ET and Q were significantly affected by forest disturbance. ET significantly decreased over the high disturbance period from 447 mm in 2000 to 427 mm in 2016, despite a 12% increase in PET over the same period. Correlation analysis showed a significant (P < 0.010) negative relationship between ET and CECA. The negative correlation was expected for ET because forest disturbance causes the mortality of forests and consequently reduced transpiration. This result is consistent with other studies that found ET was reduced after disturbance such as harvesting, fire, and insect disturbances [29, 30, 92–94]. The ET GBM model showed that after P, CECA had the next highest relative importance of 29.1%.

Table 2 also showed cumulative forest disturbance (CECA) has a positive correlation with Q and runoff ratio in the high disturbance period (2000 to 2016). This is expected as the higher levels of forest disturbance reduced ET and consequently increased the partitioning of P into Q. The significant increase of Q and runoff ratio is consistent with other studies [95–98].

It is important to mention that in this study, we used a novel approach to varying the watershed parameter, *w*, in Zhang’s equation (Eq. 1) to account for changes in ET due to forest disturbance. This approach improves our estimation of watershed-based ET which is critical for quantifying WUE. It is widely recognized that accurate estimation of watershed-based ET is challenging mainly due to the lack of widely applicable methodology [99, 100]. Many studies have investigated the long term relationship between climate and water partitioning at the watershed-level utilizing the Budyko framework [48, 101–104]. Often watershed properties are represented as a constant, such as *w* representing plant-available water capacity in Zhang’s Eq. (50) or the watershed characteristic *m* in Fuh’s Eq. (105). Increasingly, it is recognized that this static representation is not sufficient in mixed cover watersheds [106] or as forests change through time [107]. In this study, we developed a relationship between *w* and CECA based on long-term data in some comparable watersheds, and then applied this relationship to assign *w* values according to various CECA levels. The estimates of ET from this approach were checked by those calculated from the water balance method. The high consistence in ET estimates between those two methods demonstrated that our ET estimation was reliable. Additional analysis across three and five year windows was carried out to test if ET was sensitive to the assumption that the annual change in storage is zero. Results did not show significant change in either the trend or magnitude of ET, indicating that the analysis and conclusions are robust to this assumption (Additional file 1: Table S4 and Table S5). Our long term average estimated ET (471 mm) compares closely to those from similar watersheds such as Moffatt and Baker at 468 and 439 mm respectively (Additional file 1: Figure S6) [44].

### Landscape-level WUE increase under cumulative disturbance

Over the study period, we found that the average annual WUE was 0.53 g C m^**−**2^ mm^−1^ H_2_O in the Chilcotin watershed. Direct comparison with NPP values from other studies is difficult, as many are either based on other measures of carbon uptake such as Gross Primary Production (GPP), or Net Ecosystem Production (NEP), rather than NPP, measurements are from the growing season only, or taken in different climates or land covers than this study. The values of WUE that we found (~ 0.5 g C m^**−**2^ mm^−1^ H_2_O) are lower than those calculated for forests across the continental United States of America (USA)(0.93 g C kg^−1^ H_2_O) [13], which are likely more productive biomes and have very different climates. Our WUE is lower than those calculated just for the growing seasons, such as ~ 0.95 g C m^**−**2^ kg^−1^ H_2_O in European temperate evergreen conifer forest [108], but is similar to a study in North Eastern China that found annual WUE calculated as NPP/ET was in the range of 0.46–1.10 5 g C m^**−**2^ mm^−1^ H_2_O [109]. We are not aware of any other studies on annual WUE using NPP/ET in the same region as our study area.

During the high disturbance period (2000–2016), annual watershed-level WUE significantly increased in the Chilcotin watershed and correlation analysis found that WUE was significantly and positively correlated to CECA (P = 0.018). Cumulative forest disturbance played an important role in both carbon and water variables and consequently WUE changes. Additional evidence for the relationship of WUE and forest disturbance was also shown by the WUE GBM model, which found that of the independent variables we tested it with, P (55.6%) and CECA (44.4%) had the highest relative importance for predicting WUE. Further analysis using Sen’s slope, revealed that the dramatic increase in WUE (19%, P < 0.001) during the high disturbance period was mainly due to the significantly larger decrease in ET (19%) than the more resilient NPP (-2%). Clearly, this unexpected result was not only related to significant decrease in ET, but also the resilience of NPP at the large watershed scale. Increasingly, WUE is being used as a cross biome indicator of ecosystem resilience to drought, at the global scale [110] and at the basin scale across India [111] and being used to support irrigation or forest management strategies in arid regions [112]. Our finding that WUE was resilient to disturbance in the Chilcotin watershed adds to this body of work.

Studies have used WUE across large climatic gradients to assess trade offs between water use and terrestrial carbon [113, 114]. Gao et al. [113] found differences between semi-humid and arid zones in China and used this to infer optimal strategies about the location of afforestation. This suggests that our result may be area or regionally specific and underlines the importance of future studies in other geographic, climatic, or ecological types.

The majority of WUE studies are at shorter time intervals and either at the site-level or at continental or global scales [12, 13, 18, 113, 115, 116]. Stand-level studies showed WUE decreasing after some types of disturbance [25, 84, 117], although areas with substantial understory vegetation, or a low severity of disturbance often showed no change in WUE [25, 30]. Those that showed a decrease in WUE concluded that the reduction in carbon uptake was larger than the reduction in ET [117]. Contrary to this, we found that watershed-level NPP was largely sustained under high disturbance, while ET was decreased, which led to a significant increase in WUE. This result is the opposite of expected based on stand-level WUE studies, and was because NPP was sustained at the watershed-level as discussed above. This unique finding adds to the body of literature on WUE to improve our knowledge of how WUE changes with forest disturbance at the large spatial scale.

Future studies on forest disturbance should also consider the effects of climate change on WUE. Studies investigating long term trends in WUE under the effect of climate change have mixed results. Those looking at leaf or tree intrinsic WUE have often found an increase that is attributed to CO_2_ fertilization or other climate factors [26, 27]. Other studies found a decreasing WUE trend in forest biomes, or over the few last decades, compared to the decades before [14, 74, 118]. There are important regional differences to the general trends, and studies using process based models sometimes showed divergent responses [27, 74]. Although climatic variables were included in our calculations of ET and thus WUE, we did not explicitly separate out the relative role of climate change and forest disturbance in WUE variation. This will be our research priority, particularly with regard to the potential impacts of future climate change. Incorporation of full ecohydrologic modeling and independent sources of watershed-scale NPP and ET validation are also areas of high priority.

## Conclusions

Cumulative forest disturbance played an important role in both carbon and water dynamics, and consequently WUE in the Chilcotin watershed over the last 45 years. To our surprise, watershed-level forest carbon stocks and sequestration capacity were resilient after the significant cumulative disturbance, which was likely due to the initial state of the forests and varied types of disturbance at levels low enough to balance an aging carbon store with young fast growing regenerating forest. The resilient carbon variables, along with a substantial decrease in annual ET following the disturbance, drove WUE to increase by 19%. These findings have important implications for managing forest carbon and water in the large watershed or landscape scale.

## Supplementary Information

**Additional file 1.** Figures and Tables.

## Data Availability

The timeseries data for the Chilcotin watershed that supports the conclusions of this article is included within the articles additional files as Additional file 1: Table S1.

## Abbreviations

AGBIO: Above Ground Biomass
BEC: Biogeoclimatic
CECA: Cumulative Equivalent Clear-cut Area
CBM-CFS3: Carbon Budget Model of the Canadian Forest Service version 3
DOM: Dead Organic Matter
ECA: Equivalent Clear-cut Area
ET: Evapotranspiration
g C: Grams of carbon
GBM: Gradient Boosting Machine
GPP: Gross Primary Production
H2O: Water
ha: Hectare
IDF: Interior Douglas-fir
m2: Square meter
mm: Millimeter
MPB: Mountain Pine Beetle
MS: Montane Spruce
NBP: Net Biome Production
NEP: Net Ecosystem Production
NPP: Net Primary Production
P: Precipitation
PET: Potential Evapotranspiration
Q: Mean Annual Streamflow
R: The R Programming Language
RMSE: Root Mean Square Error
∆S: Annual Storage Change
SBPS: Sub-Boreal Pine–Spruce
TEC: Total Ecosystem Carbon
TIPSY: Table Interpolation Program for Stand Yields
Tmax: Annual Maximum Daily Temperature
Tmean: Annual Mean Daily Temperature
Tmin: Annual Minimum Daily Temperature
USA: United States of America
VDYP: Variable Density Yield Projection
VRI: Vegetation Resources Inventory
w: Plant-Available Water Coefficient
WUE: Water Use Efficiency
