# Supplementary material for: Dramatic increase in water use efficiency with cumulative forest disturbance at the large forested watershed scale
Source: Carbon Balance Manag. 2021 Mar 1;16:6. doi: 10.1186/s13021-021-00169-4 (PMC7923323; doi:10.1186/s13021-021-00169-4)
Supplement: Supplementary file 1 — Additional file 1. Figures and Tables. [file 13021_2021_169_MOESM1_ESM.pdf]

Supplementary Materials (CBAM-S-20-00077)

Table S1. Annual timeseries input data from 1971 – 2016 in the Chilcotin watershed, where CECA = cumulative clear-cut area, AGBIO = above ground biomass, DOM = dead organic matter, TEC = total ecosystem carbon, NPP = net primary production, NBP = net biome production, Tmean = annual average daily temperature, Tmin = annual minimum daily temperature, Tmax = annual maximum daily temperature, P = precipitation, PET = potential evapotranspiration, ET = evapotranspiration, Q = mean annual streamflow, WUE = water use efficiency.

Year	Q	CECA	Tmean	Tmin	Tmax	P	PET	ET	Runoff ratio	AGBIO	TEC	DOM	NPP	NBP	WUE
Unit	mm	%	C	C	C	mm	mm	mm	Proportion	gC m^2	gC m^2	gC m^2	gC m^2 yr^{-1}	gC m^2 yr^{-1}	gC m^2 yr^{-1} mm^{-1}
1971	160	1.1	0.51	-5.84	6.85	663	511	492	0.24	2,677	11,774	3,784	221	52	0.449
1972	168	1.2	0.02	-6.37	6.41	608	475	453	0.28	2,723	11,825	3,778	223	52	0.491
1973	133	1.3	0.67	-5.44	6.78	702	492	497	0.19	2,770	11,879	3,774	225	54	0.453
1974	176	1.3	1.25	-4.96	7.47	699	498	500	0.25	2,819	11,935	3,769	228	56	0.456
1975	151	1.4	0.18	-5.98	6.34	668	475	477	0.23	2,868	11,992	3,766	230	57	0.483
1976	199	1.4	1.27	-4.63	7.17	650	459	462	0.31	2,916	12,050	3,764	233	58	0.503
1977	158	1.4	1.15	-5.03	7.32	602	504	462	0.26	2,965	12,109	3,762	235	59	0.508
1978	161	1.5	1.06	-5.39	7.51	530	525	433	0.30	3,010	12,166	3,763	237	57	0.547
1979	140	1.6	1.07	-5.83	7.98	532	527	435	0.26	3,056	12,225	3,764	239	58	0.550
1980	155	1.7	1.42	-4.54	7.37	790	484	520	0.20	3,099	12,282	3,766	241	57	0.463
1981	175	1.8	2.32	-3.92	8.56	629	507	475	0.28	3,143	12,340	3,768	243	58	0.511
1982	178	1.9	0.51	-5.61	6.62	681	469	478	0.26	3,189	12,400	3,771	245	60	0.513
1983	147	2.0	1.58	-4.41	7.57	646	497	478	0.23	3,233	12,458	3,774	247	59	0.517

1984	151	2.2	0.98	-4.89	6.85	712	474	490	0.21	3,273	12,515	3,780	248	56	0.507
1985	130	2.7	0.57	-5.67	6.81	481	512	401	0.27	3,295	12,558	3,794	249	43	0.621
1986	160	3.3	1.79	-4.38	7.96	661	512	488	0.24	3,320	12,599	3,803	249	41	0.511
1987	170	3.7	2.83	-3.71	9.37	585	542	464	0.29	3,344	12,639	3,811	251	40	0.540
1988	133	4.3	1.86	-4.21	7.94	621	503	466	0.21	3,335	12,648	3,829	250	8	0.536
1989	148	5.1	1.71	-4.74	8.16	640	528	483	0.23	3,331	12,658	3,842	249	11	0.515
1990	177	5.7	1.65	-4.66	7.97	755	521	523	0.23	3,350	12,689	3,848	249	31	0.476
1991	275	6.2	1.99	-4.02	8.00	621	492	459	0.44	3,359	12,709	3,855	247	20	0.538
1992	205	6.8	2.65	-3.37	8.67	664	552	501	0.31	3,364	12,725	3,863	247	16	0.493
1993	148	7.3	1.63	-4.52	7.78	554	505	433	0.27	3,379	12,751	3,867	248	26	0.573
1994	181	7.9	2.03	-4.05	8.12	646	538	486	0.28	3,382	12,764	3,875	248	14	0.511
1995	162	8.3	1.85	-4.28	7.98	745	515	512	0.22	3,399	12,791	3,878	250	26	0.487
1996	187	8.8	-0.15	-6.16	5.87	731	482	489	0.26	3,405	12,807	3,884	250	16	0.512
1997	176	9.4	2.05	-3.99	8.10	793	512	523	0.22	3,408	12,820	3,891	251	13	0.479
1998	186	9.8	2.70	-3.43	8.84	646	545	486	0.29	3,412	12,834	3,897	251	14	0.516
1999	210	10.3	1.83	-4.23	7.90	721	475	480	0.29	3,416	12,847	3,903	252	13	0.525
2000	148	10.8	1.42	-4.51	7.36	615	485	447	0.24	3,410	12,850	3,911	252	4	0.564
2001	142	11.1	1.61	-4.41	7.64	638	480	453	0.22	3,432	12,879	3,910	253	28	0.558
2002	150	11.1	1.59	-4.65	7.84	522	497	410	0.29	3,470	12,923	3,905	256	45	0.623
2003	169	11.1	2.05	-4.40	8.50	620	530	467	0.27	3,307	12,941	4,113	248	18	0.531
2004	173	14.9	2.63	-3.66	8.92	727	557	514	0.24	3,285	12,961	4,157	250	20	0.486
2005	186	15.9	2.28	-3.94	8.50	671	524	477	0.28	3,277	12,984	4,187	251	23	0.527
2006	157	17.3	2.21	-4.52	8.94	609	549	459	0.26	3,306	13,006	4,172	254	22	0.554
2007	185	19.5	1.56	-4.53	7.65	699	505	470	0.27	3,194	13,004	4,301	251	-1	0.534
2008	144	22.1	1.08	-5.38	7.54	615	487	429	0.23	3,212	13,012	4,284	255	8	0.593
2009	146	29.3	1.13	-5.66	7.91	590	543	428	0.25	2,918	12,815	4,442	240	-197	0.561
2010	185	31.4	2.14	-4.05	8.33	624	523	428	0.30	2,927	12,813	4,428	242	-2	0.565

2011	198	33.4	1.02	-4.92	6.96	659	459	403	0.30	2,966	12,832	4,397	243	18	0.605
2012	177	35.3	1.50	-4.72	7.72	620	510	412	0.29	3,007	12,853	4,367	247	22	0.600
2013	168	36.9	2.15	-4.16	8.45	521	534	381	0.32	2,968	12,846	4,404	246	-7	0.645
2014	159	38.0	1.96	-4.46	8.37	608	546	415	0.26	2,974	12,861	4,409	247	14	0.595
2015	199	38.8	3.02	-3.44	9.48	500	580	381	0.40	3,016	12,887	4,380	250	26	0.657
2016	192	39.6	2.66	-3.53	8.85	661	545	427	0.29	3,066	12,922	4,351	254	36	0.595

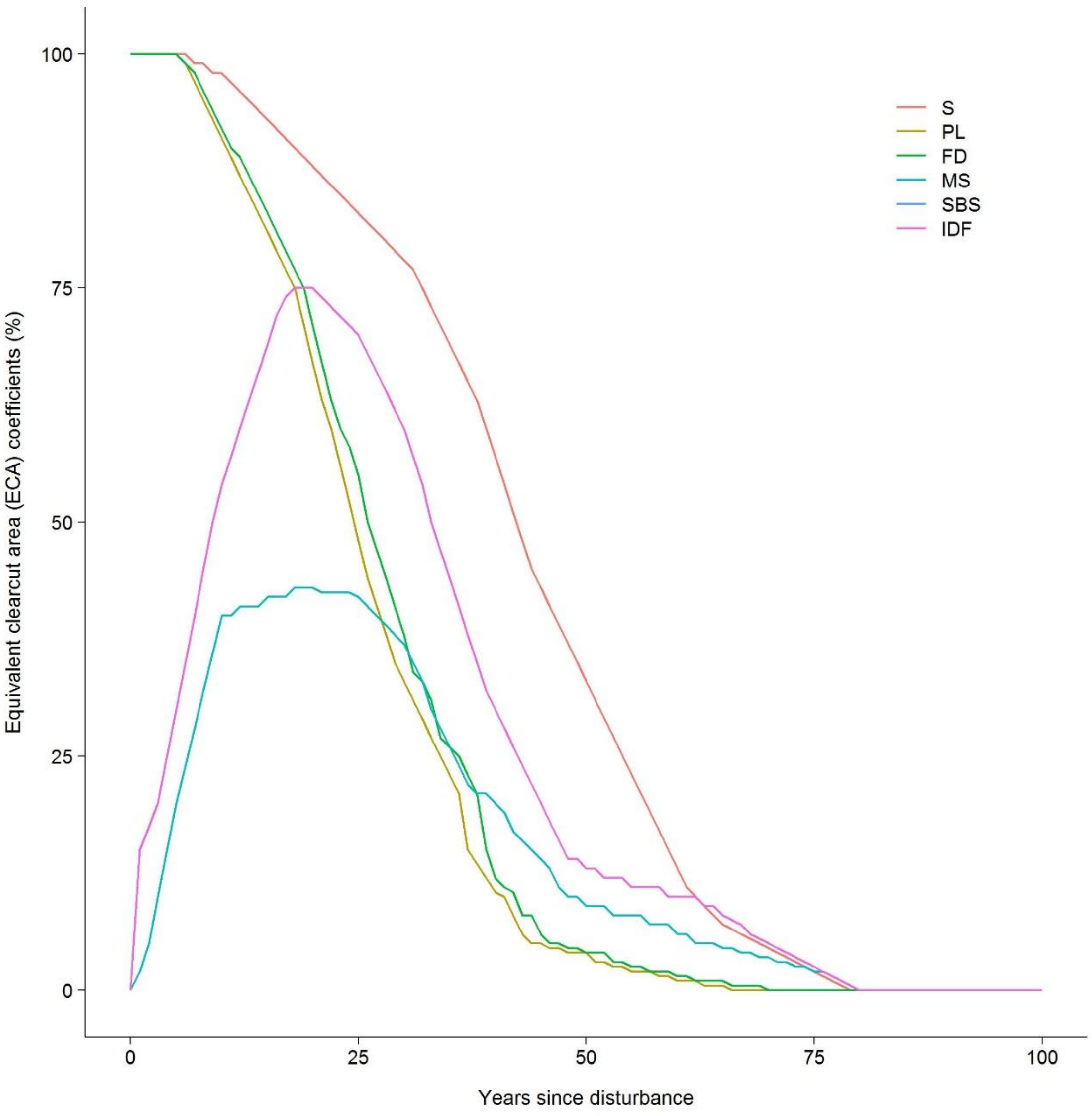

Figure S1. Annual generalized Cumulative Equivalent Clear-cut Area (CECA) curves by disturbance type since disturbance occurred for 100 years, where clear-cut CECA leading species is either S = spruce, PL = Lodgepole pine, FD = Douglas fir, and mountain pine beetle biogeoclimatic zone is either MS = Montane Spruce, SBS = Sub-Boreal Spruce, or IDF = Interior Douglas-fir.

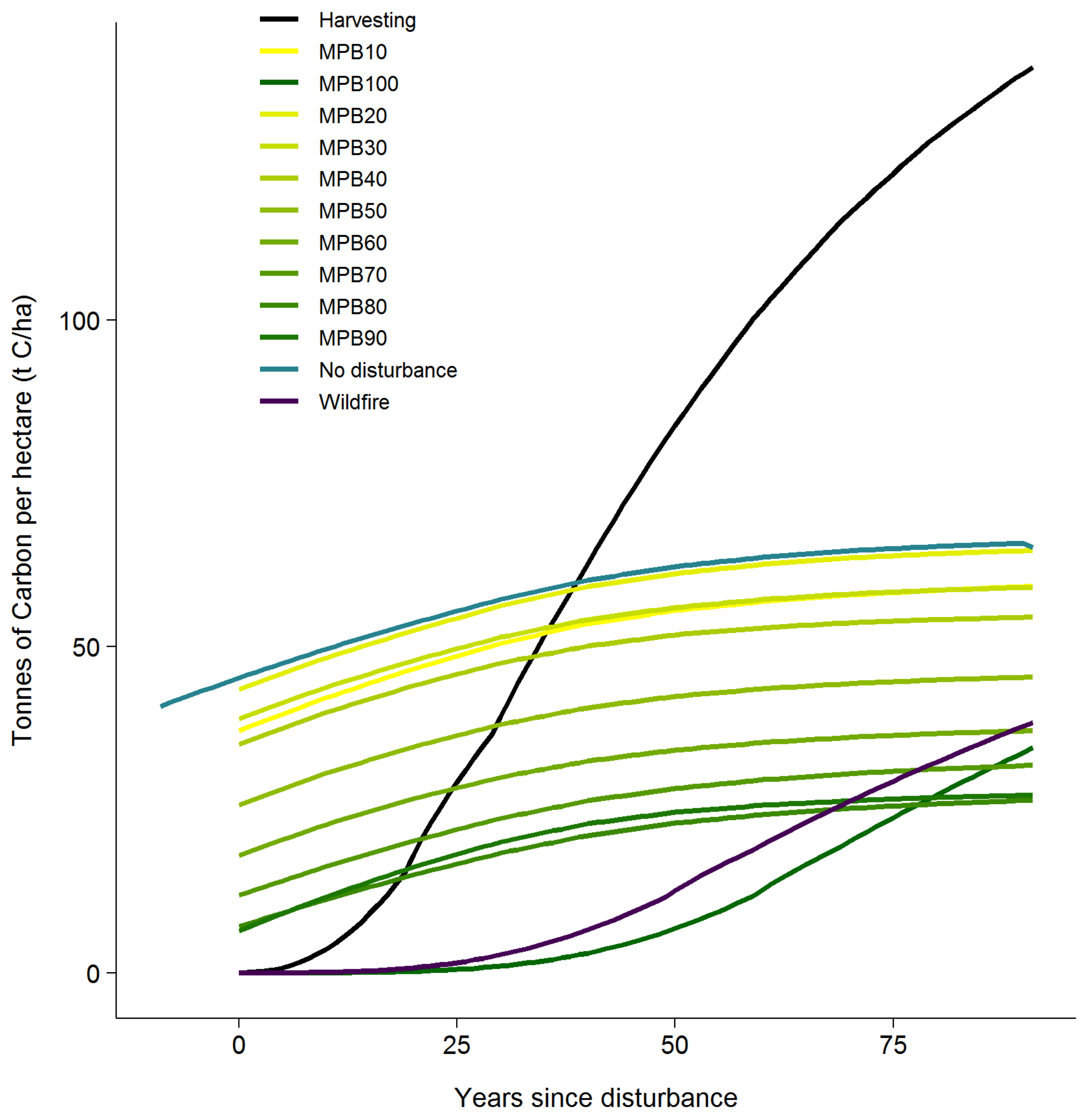

Figure S2. Above ground biomass (AGBIO) curves by disturbance type since disturbance occurred, area weight averaged across all forest types in the Chilcotin watershed for 100 years.

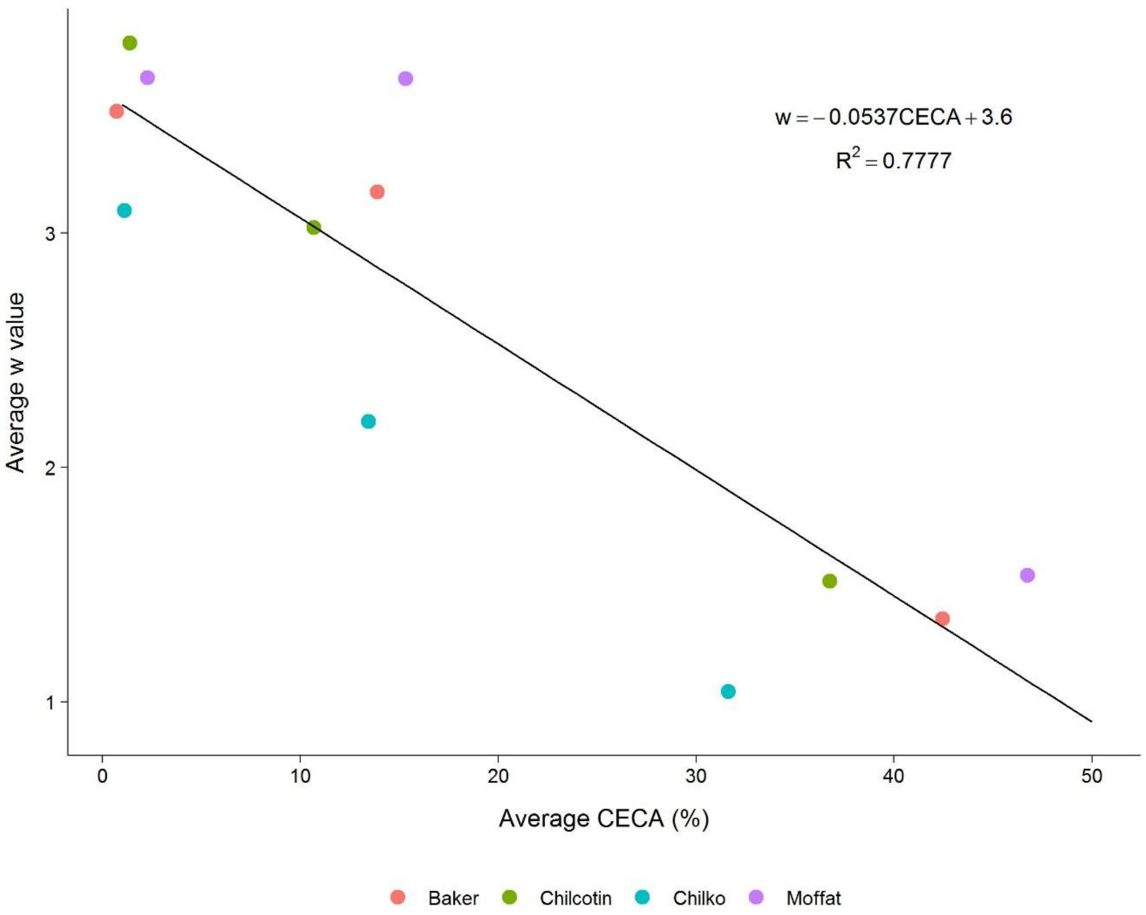

Figure S3. Grouped low (0 – 5), moderate (10 – 20), and high (> 30) cumulative equivalent clear-cut area (CECA) and average *w* values from selected similar watersheds in the central interior of British Columbia (Baker, Chilcotin, Chilko, and Moffat). The best fit linear regression that describes the relationship between CECA and w is shown as a black line.

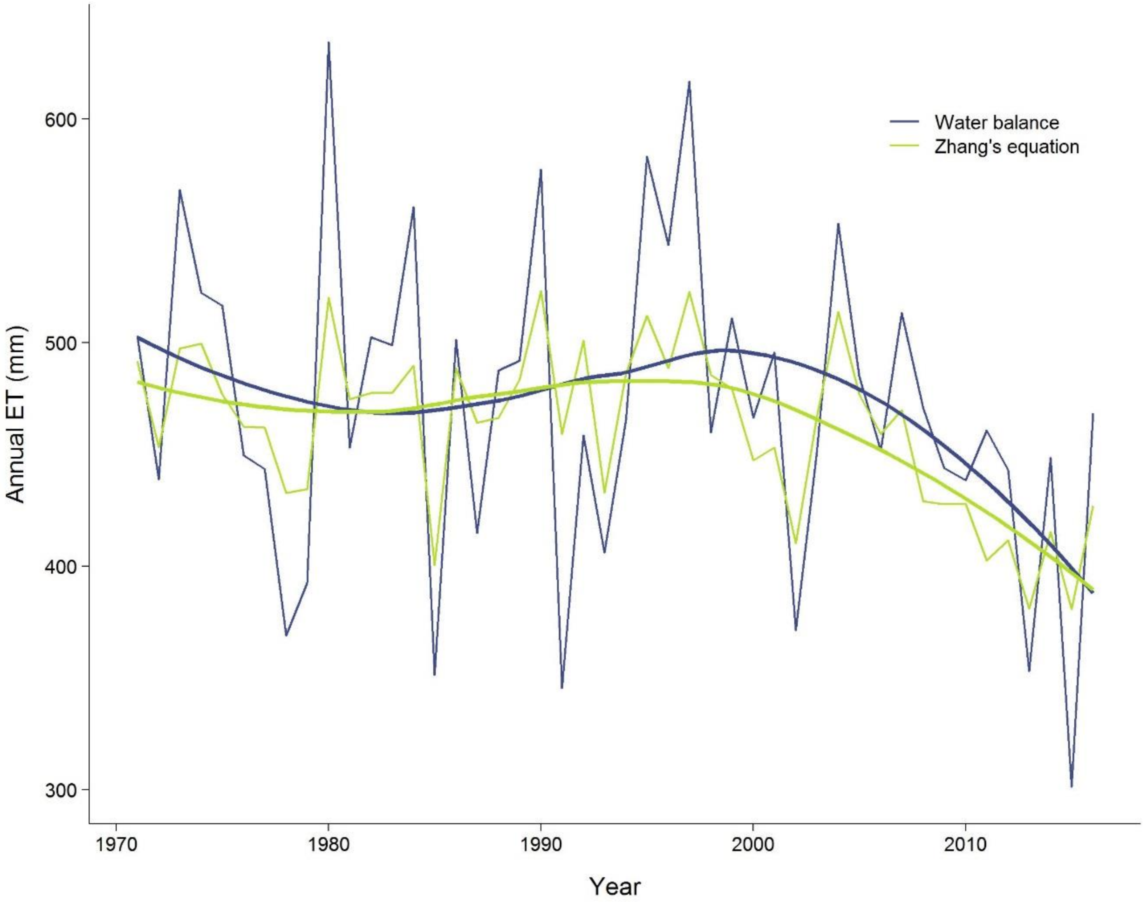

Figure S4. Watershed-level evapotranspiration (ET) in the Chilcotin watershed from 1971 – 2016 calculated using the water balance method or Zhang's equation with '*w*' varying based on Cumulative Equivalent Clear-cut Area (CECA). The thick lines are smoothed using Loess regression.

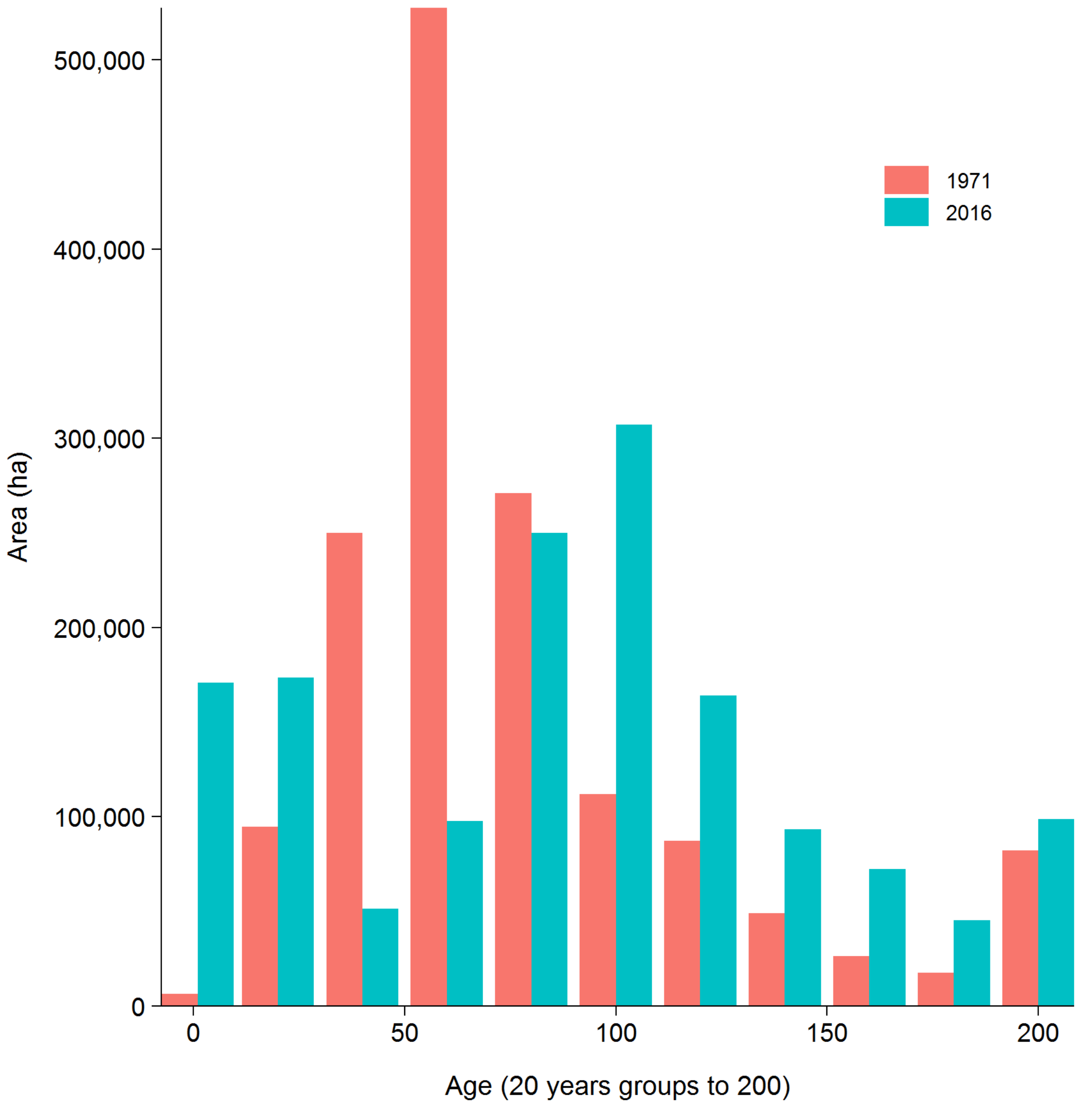

Figure S5. Age class distribution of the forests in the Chilcotin watershed in 1971 and 2016, where age is grouped to the nearest 20 up to 200 years.

Table S2. Disturbance, species and other parameters used in CBM-CFS3

Disturbance code	Mapped CBM-CFS3 disturbance type	
fire	Wildfire	
harv	Clear-cut with slash-burn	
mpb5	Generic 5% mortality	
mpb10	Generic 10% mortality	
mpb15	Generic 15% mortality	
mpb20	Generic 20% mortality	
mpb25	Generic 25% mortality	
mpb30	Generic 30% mortality	
mpb35	Generic 35% mortality	
mpb40	Generic 40% mortality	
mpb45	Generic 45% mortality	
mpb50	Generic 50% mortality	
mpb55	Generic 55% mortality	
mpb60	Generic 60% mortality	
mpb65	Generic 65% mortality	
mpb70	Generic 70% mortality	
mpb75	Generic 75% mortality	
mpb80	Generic 80% mortality	
mpb85	Generic 85% mortality	
mpb90	Generic 90% mortality	
mpb95	Generic 95% mortality	
mpb100	Insect disturbance	
**Other parameters**		
Ecozone	Montane Cordillera	
Administrative boundary	British Columbia	
Regeneration delay	0	
UNFCCC land class	0 (managed forest or forest land remaining forest land)	
Historical disturbance type	Wildfire	
**Species code**	**Mapped CBM-CFS3 species**	**Hard/soft wood**
AC	Balsam poplar	Hardwood
ACT	Black cottonwood	Hardwood
AT	Trembling aspen	Hardwood
BL	Balsam fir	Softwood
C	Cedar	Softwood
CW	Western redcedar	Softwood
EP	Birch	Hardwood
FD	Douglas-fir - Genus type	Softwood

FDI	Douglas-fir - Genus type	Softwood
HW	Western hemlock	Softwood
PL	Lodgepole pine	Softwood
PLI	Lodgepole pine	Softwood
S	Spruce - Genus type	Softwood
SB	Black spruce	Softwood
SE	Engelmann spruce	Softwood
SW	White spruce	Softwood
SX	Spruce - Genus type	Softwood

Table S3. Description of CBM-CFS3 pools

Variable	Description of variable	Unit	CBM pools/query
AGBIO	Carbon stored in above ground biomass	Tonnes of carbon	Softwood merchantable + hardwood merchantable + softwood sub-merchantable + hardwood sub-merchantable + softwood other + hardwood other + softwood foliage + hardwood foliage
BGBIO	Carbon stored in below ground biomass	Tonnes of carbon	Softwood coarse roots + hardwood coarse roots + softwood fine roots + softwood coarse roots
DOM	Carbon stored in dead organic matter	Tonnes of carbon	Aboveground very fast soil + aboveground fast soil + aboveground slow soil + belowground fast soil + medium soil + softwood stem snags + softwood branch snags + hardwood stem snags + hardwood branch snags
Soil	Carbon stored in the soil	Tonnes of carbon	Belowground very fast soil+ belowground slow soil + black carbon + peat
TEC	Total ecosystem carbon. The sum of all pools.	Tonnes of carbon	AGBIO + BGBIO + DOM + soil
NPP	Net primary production. The net annual amount of carbon gains into the watershed's ecosystem	Tonnes of carbon per hectare per year	Aboveground biomass increment + merchantable litter input + sub-merchantable litter input + foliage litter input + other litter input + belowground biomass increment + coarse litter input + fine litter input Where disturbance in that year is zero

NBP	Net biome production. The year to year change in carbon stocks after accounting for losses from disturbances such as fire, logging and MPB. An appropriate measure of system carbon balances over longer period of time.	Tonnes of carbon per hectare per year	Aboveground biomass increment + merchantable litter input + sub-merchantable litter input + foliage litter input + other litter input + belowground biomass increment + coarse litter input + fine litter input – softwood production – hardwood production – biomass CO2 emissions – biomass CH4 emissions – biomass CO emissions – DOM CO2 emissions – DOM CH4 emissions – DOM CO emissions – DOM production<br>Where land conversion does not occur

Table S4. Comparison of evapotranspiration (ET) calculated using Zhang's equation and runoff ratio across 1, 3, and 5 year intervals in the Chilcotin watershed from six selected points

		Number of years		
**Variable**	**Period**	**1**	**3**	**5**
**ET**	Reference	455	459	466
			1%	3%
	Disturbance	436	435	438
			0%	1%
	All	445	447	452
			0%	2%
**Runoff ratio**	Reference	0.28	0.29	0.28
			4%	-2%
	Disturbance	0.26	0.27	0.27
			4%	3%
	All	0.27	0.28	0.27
			4%	1%

Table S5. Comparison of trend analysis for evapotranspiration (ET) estimates using Zhang's equation across 1, 3, and 5 year intervals in the Chilcotin watershed from 1971 – 2016

**Interval**	**1971 - 1999**		**2000 - 2016**	
	**Tau**	**P-value**	**Tau**	**P-value**
1	0.164	0.228	-0.417	**0.027**
3	0.222	0.466	-0.800	0.086
5	0.600	0.221	-0.333	1.000

Figure S6. Comparison of long term evapotranspiration (ET) and run-off ratio (annual streamflow divided by annual precipitation) from the Chilcotin and other comparable watershed studies

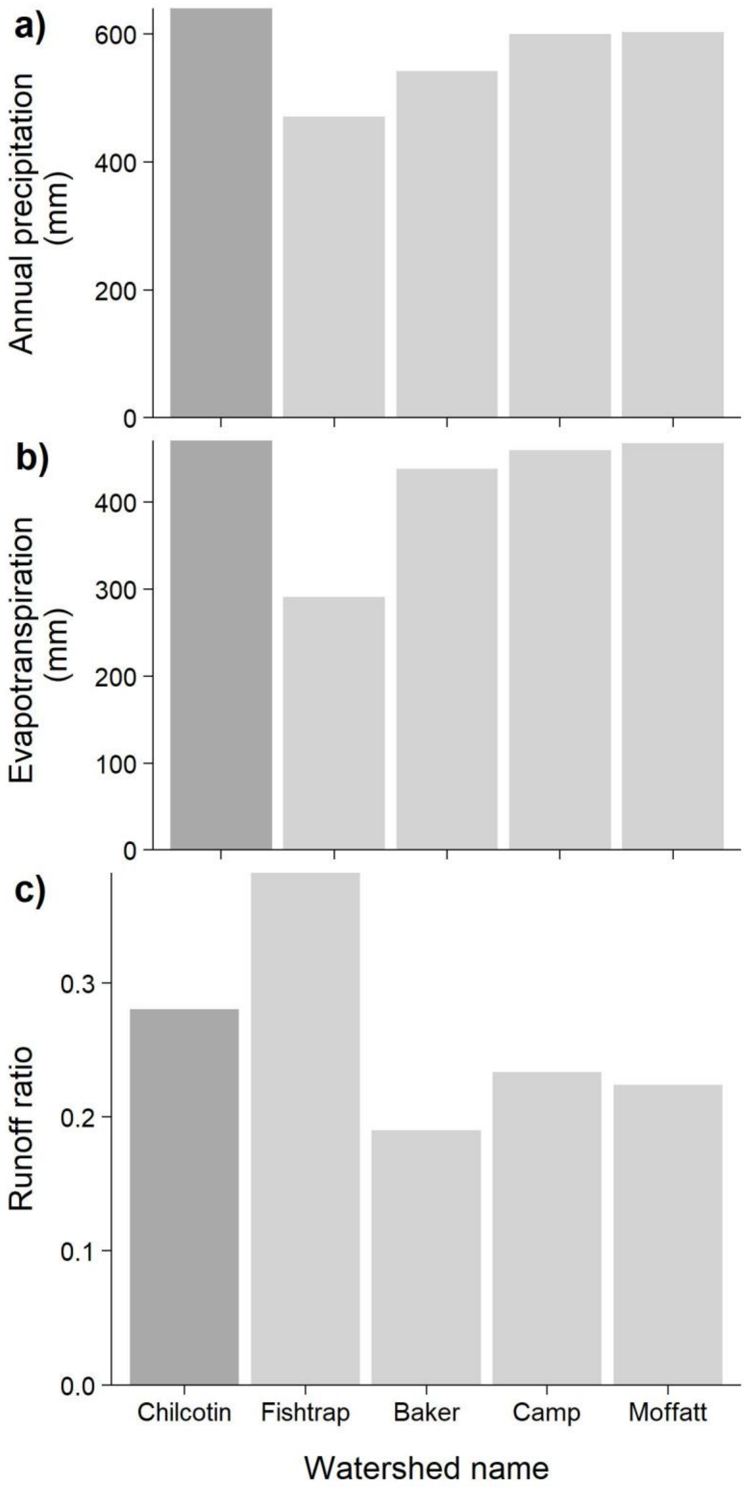

Sources:

Camp Creek: Moore, R. D., & Scott, D. F. (2005). Camp Creek Revisited: Streamflow Changes Following Salvage Harvesting in a Medium-Sized, Snowmelt-Dominated Catchment. Canadian Water Resources Journal / Revue canadienne des ressources hydriques, 30(4), 331-344. doi:10.4296/cwrj3004331

Fishtrap Creek: Winkler, R. D., Moore, R. D., Redding, T. E., Spittlehouse, D. L., Smerdon, B. D., & Carlyle-Moses, D. E. (2010). The Effects of Forest Disturbance on Hydrologic Processes and Watershed Response (Chapter 7). Paper presented at the Compendium of Forest Hydrology and Geomorphology in British Columbia, Victoria, British Columbia, Canada.

Baker: Zhang, M., & Wei, X. (2012). The effects of cumulative forest disturbance on streamflow in a large watershed in the central interior of British Columbia, Canada. Hydrology and Earth System Sciences, 16, 2021-2034. doi:doi:10.5194/hess-16-2021-2012

Moffatt: Zhang, M., Wei, X., & Li, Q. (2017). Do the hydrological responses to forest disturbances in large watersheds vary along climatic gradients in the interior of British Columbia, Canada? Ecohydrology, 10(2), e1840. doi:10.1002/eco.1840

Figure S7. Comparison of modelled net merchantable volume and ground plot data in the Chilcotin watershed

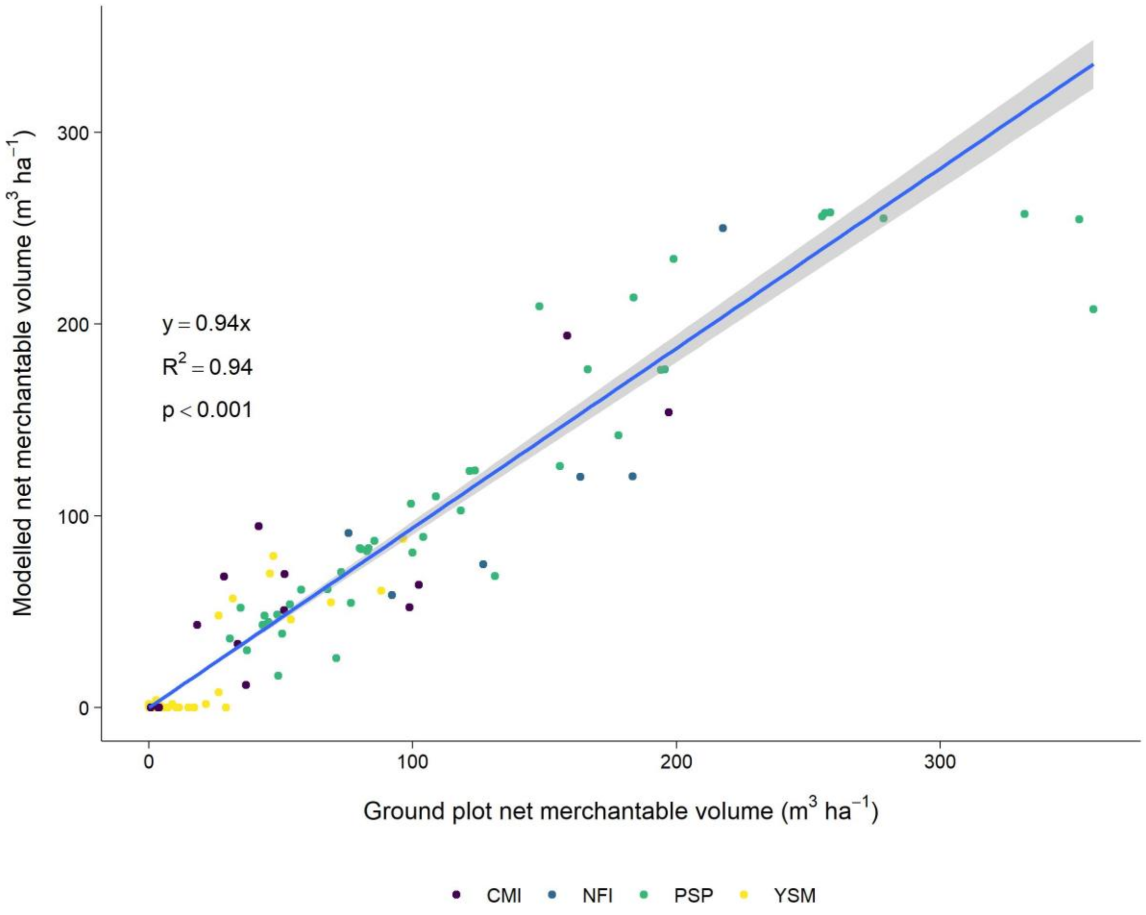

Where the point colour shows the type of ground sample, which is one of CMI=change monitoring inventory, NFI=National Forest Inventory, PSP=permanent sample plots, or YSM=young stand monitoring. Accessed from: https://bcgov-env.shinyapps.io/ground_sample_deploy/ on 16 December 2020
